# Is It Possible to Predict Weight Loss After Bariatric Surgery?—External Validation of Predictive Models

**DOI:** 10.1007/s11695-021-05341-w

**Published:** 2021-03-13

**Authors:** Izabela A. Karpińska, Jan Kulawik, Magdalena Pisarska-Adamczyk, Michał Wysocki, Michał Pędziwiatr, Piotr Major

**Affiliations:** 1 Students’ Scientific Group at 2nd Department of Surgery, Jagiellonian University Medical College, Jakubowskiego 2 st., 30-688 Krakow, Poland; 2 2nd Department of General Surgery, Jagiellonian University Medical College, Jakubowskiego 2 st., 30-688 Krakow, Poland; 3 Department of General Surgery and Surgical Oncology, Ludwik Rydygier Memorial Hospital in Cracow, Krakow, Poland; 4 Centre for Research, Training and Innovation in Surgery (CERTAIN Surgery), Jakubowskiego 2 st., 30-688 Krakow, Poland

**Keywords:** Risk prediction models, External validation, Weight loss, Bariatric surgery

## Abstract

**Background:**

Bariatric surgery is the most effective obesity treatment. Weight loss varies among patients, and not everyone achieves desired outcome. Identification of predictive factors for weight loss after bariatric surgery resulted in several prediction tools proposed. We aimed to validate the performance of available prediction models for weight reduction 1 year after surgical treatment.

**Materials and Methods:**

The retrospective analysis included patients after Roux-en-Y gastric bypass (RYGB) or sleeve gastrectomy (SG) who completed 1-year follow-up. Postoperative body mass index (BMI) predicted by 12 models was calculated for each patient. The correlation between predicted and observed BMI was assessed using linear regression. Accuracy was evaluated by squared Pearson’s correlation coefficient (*R*^2^). Goodness-of-fit was assessed by standard error of estimate (SE) and paired sample *t* test between estimated and observed BMI.

**Results:**

Out of 760 patients enrolled, 509 (67.00%) were women with median age 42 years. Of patients, 65.92% underwent SG and 34.08% had RYGB. Median BMI decreased from 45.19 to 32.53kg/m^2^ after 1 year. EWL amounted to 62.97%. All models presented significant relationship between predicted and observed BMI in linear regression (correlation coefficient between 0.29 and 1.22). The best predictive model explained 24% variation of weight reduction (adjusted *R*^2^=0.24). Majority of models overestimated outcome with SE 5.03 to 5.13kg/m^2^.

**Conclusion:**

Although predicted BMI had reasonable correlation with observed values, none of evaluated models presented acceptable accuracy. All models tend to overestimate the outcome. Accurate tool for weight loss prediction should be developed to enhance patient’s assessment.

**Graphical abstract:**

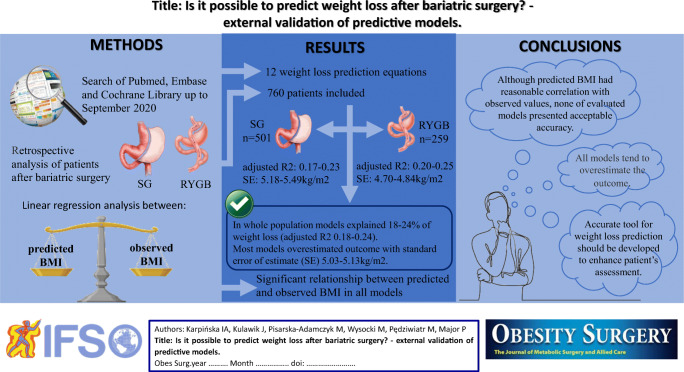

**Supplementary Information:**

The online version contains supplementary material available at 10.1007/s11695-021-05341-w.

## Introduction

Bariatric surgery, while not the first-line therapy, was proved to be the most effective means of achieving sustained weight loss in morbidly obese patients in comparison to non-operative treatment [1]. Although several surgical techniques are currently available, two most commonly performed are sleeve gastrectomy (SG) and Roux-en-Y gastric bypass (RYGB) [2]. Selection of the proper bariatric procedure is subjective and has always been an area of active debate.

Despite comprehensive preoperative assessment of each candidate, weight loss outcomes after intervention show distinct deviation ranging from 37.6 to 94.4% of excessive weight loss (EWL) [1]. More importantly, 7 up to 25% of bariatric patients fail to accomplish optimal result, defined as above 50% of EWL [1].

Understanding of possible weight loss results of bariatric treatment would facilitate preoperative patient’s assessment and decision-making process [3]. It would optimize selection of candidates more likely to benefit from the surgery and provide them with the appropriate procedure, resulting in better long-term effects on sustained weight loss and obesity-related comorbidities [1, 4, 5]. Therefore, reasonable estimation of expected outcomes after bariatric surgery, especially weight loss, seems to be crucial not only for surgical candidates but also their physicians.

Numerous studies revealed that weight loss after bariatric treatment depends on various factors including demographic aspects, comorbidity rate, psychological profile, lifestyle, or socioeconomic status [6–11]. However, it is difficult to take all of them into account during preoperative assessment of the patient in the proportion each one contributes to the outcome. It would, therefore, be helpful to have all significant predictors of weight loss integrated into easy-to-use estimation tool.

With constant development of bariatric surgery, there has been increasing focus on inventing adequate tools for outcomes prediction [12]. As a result, more and more models and scoring systems for the assessment of postoperative weight loss or comorbidities alleviation are being proposed [12, 13]. Currently the number of published weight loss prediction models becomes overwhelming. Moreover, most of them require external validation and precise statistical tools assessment. Still, there is lack of comprehensive scientific conclusion on the effectiveness of existing tools in predicting weight loss after bariatric surgery and their utility in clinical practice. Thus, we designed a study to perform a systematic review of the literature for the identification of available models and validate them as the predictors of weight loss at 1 year after SG or RYGB as well as compare their accuracy.

## Materials and Methods

### Study Design

In this retrospective cohort study, we performed systematic review of the literature to identify predictive models for weight loss after bariatric surgery. The predicted postoperative BMI was calculated for each patient according to original equations based on data obtained from medical records. Then, the relationship between predicted and observed BMI was assessed.

### Study Population

We included consecutive patients admitted to our department between April 2009 and October 2017 who underwent either SG or RYGB and completed 1 year of postoperative follow-up. Patients with initially incomplete data, body mass index (BMI) under 30 kg/m^2^, and revisional surgery were excluded from the analysis.

We divided study population into 3 groups: the ALL group including patients after SG and RYGB, the RYGB group including patients after RYGB, and the SG group including patients after SG.

Candidates for bariatric surgery were evaluated by a multidisciplinary team of surgeons, dieticians, psychologists, clinical nurse specialists, and anesthetists. Demographic, anthropometric, and clinical data were recorded pre- and postoperatively. The follow-up schedule comprised appointment at 1 year after surgery.

### Surgical Techniques

All participants underwent either laparoscopic SG or laparoscopic RYGB. Each patient was qualified for the appropriate type of procedure in accordance with the Polish Guidelines for Metabolic and Bariatric Surgery [14]. The surgical techniques used in our department have been described in detail in our previous publications [15, 16]. During laparoscopic SG, a 34-French gastric bougie was used to calibrate the gastric sleeve. Gastrectomy started 4–5 cm proximal to the pylorus with continuously applied linear staplers straight to the angle of His. The length of alimentary and enzymatic limb during RYGB was standardized in all patients, 150 and 100 cm, respectively.

### Data Collection

Sex, age, height, weight, BMI, comorbidities, preoperative weight loss, time of the procedure, and length of hospital stay (LOS) were collected retrospectively from medical histories. Age was calculated as the difference between the date of birth and the date of surgery. BMI was calculated from the weight (in kilograms) and divided by the square of height (in meters). Investigated comorbidities included diagnosis of hypertension (HTN), heart disease (defined as coronary artery disease or past myocardial infarction), type 2 diabetes mellitus (T2DM), metabolic syndrome, hyperlipidemia, kidney disease, liver disease, obstructive sleep apnea (OSA), polycystic ovary syndrome (PCOS), gastroesophageal reflux disease (GERD), and arthritis.

### Weight Loss After Bariatric Surgery

Evaluated outcome of bariatric treatment was defined as patient’s weight at 1 year after initial procedure, assessed by postoperative BMI. Weight change was expressed using percentage weight loss (WL), percentage EWL, and percentage excessive body mass index loss (EBMIL) obtained according to the previously described formulas [17]. Ideal body weight was calculated as equivalent to BMI 25 kg/m^2^. Adequate weight loss after intervention was defined as above 50% EWL [18].

### Model Selection

Literature search was performed in accordance with Preferred Reporting Items for Systematic Reviews and Meta-Analysis (PRISMA) recommendations [19]. Search of PubMed, Embase, and Cochrane Library databases were performed on September 18, 2020. The following search terms were used: bariatric surgery, postoperative weight loss, weight loss prediction, and prediction model. Additionally, the articles’ reference lists were searched manually for further studies. At first titles and abstracts of each identified study were evaluated, and then full texts for potentially relevant articles were assessed. We included English written studies, investigating all types of bariatric surgery, with prospective and retrospective design which attempted to create an individualized prediction model for postoperative weight loss. Papers presenting models based on non-individualized or postoperative factors as well as variables not routinely checked in our daily practice were excluded.

### Statistical Analysis

Continuous variables are presented as mean and standard deviation (SD) or median and interquartile range (IQR) for normally and non-normally distributed variables, respectively. Categorical variables are presented as numbers and percentages. To confirm the normality of the distribution of the continuous variables, we used the Shapiro-Wilk and the Kolmogorov-Smirnov with the Lilliefors correction tests. Equality of variances was assessed using the Brown-Forsythe test. Comparison between RYGB and SG groups was established using an independent *t* test or Mann–Whitney *U* test as appropriate for continuous variables and Chi-square test with Fisher’s correction for categorical variables. Comparison of clinical data before and after surgery was performed with the use of a paired sample *t* test or Wilcoxon test adequately.

The predicted postoperative BMI was calculated for each patient according to the original equations. If model was designed to predict weight change measures other than BMI, it was obtained with mathematical conversions. The relationship between predicted and observed BMI was assessed with linear regression method. Correlation parameters included regression coefficient (B) with 95% confidence interval (95% Cl). To evaluate the diagnostic accuracy of each model, adjusted squared Pearson’s correlation coefficient (*R*^2^) was used. Calibration was assessed by standard error of the estimate (SE) and root mean square error (RMSE) as well as paired sample *t* test between mean predicted and mean observed BMI. Good calibration was indicated by *p*>0.05. Additionally, the difference between mean predicted and mean observed BMI was obtained.

For all inferential statistics, statistical significance was defined as *p*≤0.05. All calculations were done with STATISTICA 13.0 software (StatSoft Inc., Tulsa, OK, USA).

## Results

### Study Recruitment

**Fig. 1:**
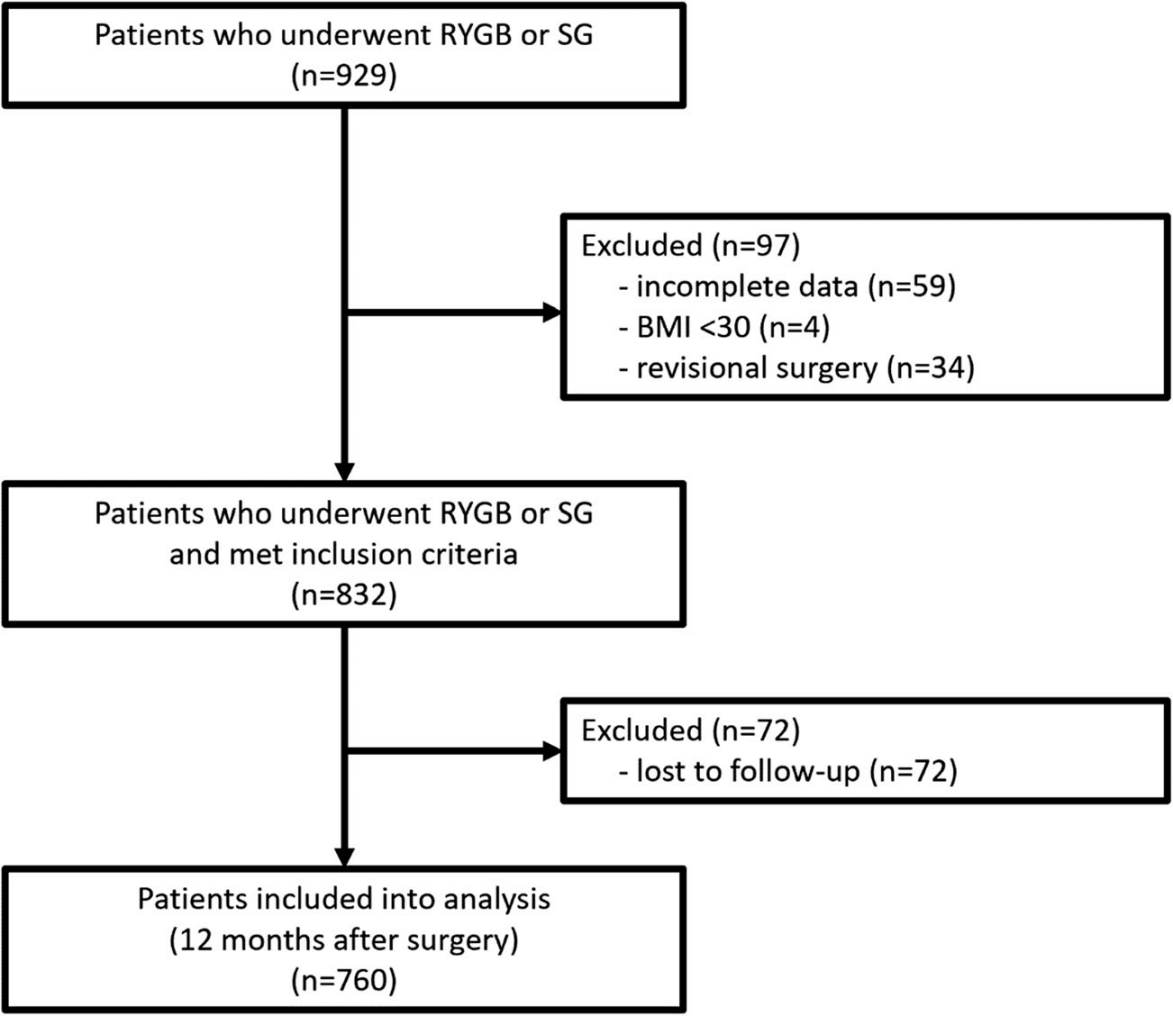
Patients flow through the study. Abbreviations: RYGB, Roux-en-Y gastric bypass; SG, sleeve gastrectomy; BMI, body mass index

A total of 929 patients underwent SG or RYGB in our department from April 2009 to October 2017. Ninety-seven (10.44%) patients were excluded because they did not meet the inclusion criteria. Subsequently, 72 patients (8.65%) were excluded on account of loss to follow-up. Ultimately, the study sample comprised 760 patients (Fig. 1).

### Baseline Characteristics and Outcome

Out of 760 patients enrolled in our study, 509 (66.71%) were women whereas 251 (32.90%) were men with median age 42 years. On average patients managed to accomplish 4 kg preoperative weight loss. The most common comorbidities were metabolic syndrome, HTN, hyperlipidemia, and T2DM. The majority of patients underwent SG. Average LOS was 4 days (Table 1). Both weight and BMI decreased significantly 1 year after intervention (Supplementary table 1). Most of participants achieved adequate weight loss after surgery with median EWL reaching 62.56% (Table 2). Detailed patient characteristics are listed in Tables 1 and 2. Comparison of SG and RYGB groups is provided in Tables 1, 2, and Supplementary table 1.

**Table 1:** Characteristics of the study population and comparison of characteristics between RYGB and SG groups

Variable	ALL (*n*=760)	RYGB (*n*=259)	SG (*n*=501)	*p*-value
Demographics				
Age, years	42.00 (16.00)	46.00 (15.00)	40.00 (16.00)	**<0.0001**
Height, cm	170.00 (12.00)	170.00 (11.00)	170.00 (12.00)	0.60
Weight, kg	130.00 (30.00)	131.00 (30.50)	130.00 (29.00)	0.11
BMI, kg/m^2^	46.00±6.52	46.36±6.65	45.81±6.44	0.12
Gender				
Women	509 (66.71)	162 (62.55)	347 (69.26)	0.06
Men	251 (32.90)	97 (37.45)	154 (30.74)	0.06
Comorbidities				
HTN	503 (65.92)	193 (74.52)	310 (61.88)	**0.0005**
Heart disease	49 (6.42)	23 (8.88)	26 (5.19)	0.05
T2DM	260 (34.08)	140 (54.05)	120 (23.95)	**<0.0001**
Metabolic syndrome	580 (76.02)	189 (72.97)	391 (78.04)	0.12
Hyperlipidemia	309 (40.50)	148 (57.14)	161 (32.14)	**<0.0001**
Kidney disease	7 (0.92)	3 (1.16)	4 (0.80)	**<0.0001**
Liver disease	88 (11.53)	35 (13.51)	53 (10.58)	0.22
OSA	92 (12.06)	39 (15.06)	53 (10.58)	0.07
PCOS	30 (3.93)	8 (3.09)	22 (4.39)	**<0.0001**
GERD	64 (8.39)	32 (12.36)	32 (6.39)	**0.005**
Arthritis	151 (19.79)	52 (20.08)	99 (19.76)	0.92
Perioperative data				
Preoperative WL, kg	4.00 (8.00)	5.00 (10.00)	3.00 (8.00)	**0.04**
Time of the procedure, min	110.00 (60.00)	135.00 (70.00)	95.00 (45.00)	**<0.0001**
LOS, days	4.00 (2.00)	4.00 (2.00)	4.00 (2.00)	**0.03**

Data are shown as mean ± standard deviation or median (interquartile range) for continuous variables and as number (percentage) for categorical variables

*p*-values refer to the comparison between RYGB and SG groups with the use of an independent *t* test or Mann–Whitney *U* test for continuous variables and Chi-square test with Fisher’s correction for categorical variables

Embolden *p*-values indicate statistically significant result

Abbreviations: *RYGB* Roux-en-Y gastric bypass, *SG* sleeve gastrectomy, *BMI* body mass index, *HTN* hypertension, *T2DM* type 2 diabetes mellitus, *OSA* obstructive sleep apnea, *PCOS* polycystic ovary syndrome, *GERD* gastroesophageal reflux disease, *WL* weight loss, *LOS* length of hospital stay, *EWL* excess weight loss, *EBMIL* excess body mass index loss

**Table 2:** Postoperative outcome at 1 year in the study population and comparison of outcomes between RYGB and SG groups

Variable	ALL (*n*=760)	RYGB (*n*=259)	SG (*n*=501)	*p*-value
Weight, kg	94.79 (19.00)	96.79 (17.00)	92.79 (17.00)	**0.0006**
BMI, kg/m^2^	32.82±5.76	33.59±5.40	32.42±5.90	**0.002**
WL, %	28.02±11.89	26.82±11.30	28.64±12.15	**0.045**
EWL, %	62.56 (33.49)	58.06 (30.46)	65.55 (26.59)	**0.007**
>50% EWL	523 (68.82)	169 (65.25)	354 (70.66)	**<0.0001**
EBMIL, %	62.56 (33.49)	58.06 (30.46)	65.55 (26.59)	**0.007**

Data are shown as mean ± standard deviation or median (interquartile range) for continuous variables and as number (percentage) for categorical variables

*p*-values refer to the comparison between RYGB and SG groups with the use of an independent *t* test or Mann–Whitney *U* test for continuous variables and Chi-square test with Fisher’s correction for categorical variables

Embolden *p*-values indicate statistically significant result

Abbreviations: *RYGB* Roux-en-Y gastric bypass, *SG* sleeve gastrectomy, *BMI* body mass index, *WL* weight loss, *EWL* excess weight loss, *EBMIL* excess body mass index loss

### Model Selection

**Fig. 2:**
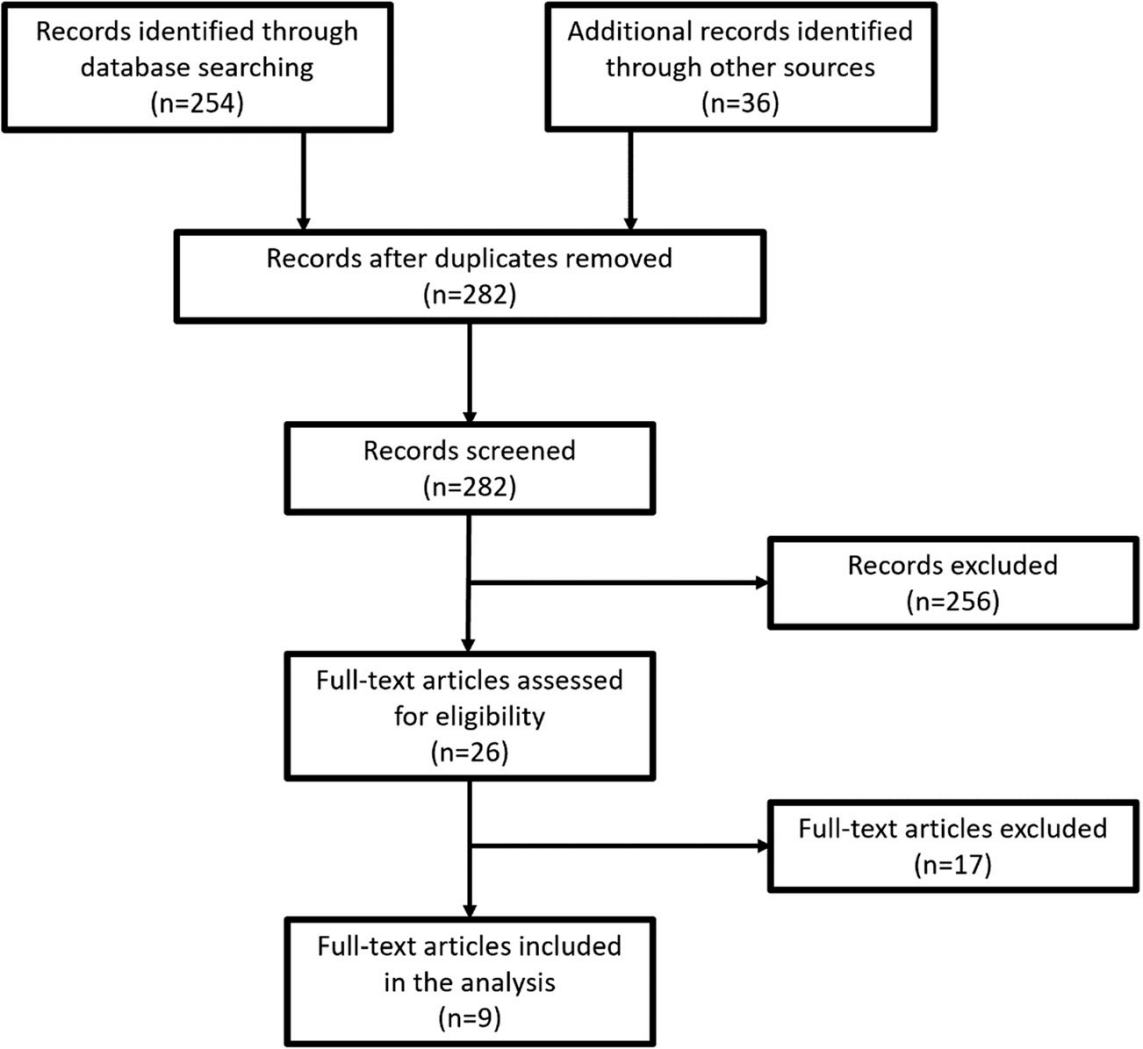
The process of study selection

Out of 282 results primarily identified by the literature search, 9 studies met eligibility criteria and were included in the analysis [3, 20–27]. Detailed process of study selection is shown on Fig. 2.

Baltasar et al. proposed four different models for weight loss prediction. The first one was designed to predict BMI after duodenal switch, and for the purpose of our study, it was named Baltasar_1 [20]. In 2011 Baltasar et al. proposed three subsequent models for BMI prediction referring to all bariatric population (Baltasar_2) as well as different procedures (Baltasar_3 for RYGB and Baltasar_4 for SG) [21]. Similarly, Cottam et al. proposed two equations with regard to predict %EWL (Cottam_1) and BMI reduction (Cottam_2) but on SG population [3]. There were four models derived from RYGB cohorts. Wood predicts BMI whereas Wise assesses EBMIL [22, 23]. Seyssel and Velázquez-Fernández focus on WL evaluation [25, 27]. On the other hand, Goulard and Janik were designed to predict BMI after SG [24, 26]. Consequently, there are 12 various weight loss prediction equations ultimately incorporated into our study.

All evaluated models were developed between 2009 and 2019 with follow-up period from 0.5 up to 3 years. The formulas of each prediction model and characteristics of training cohorts are provided in Table 3.

**Table 3:** The overview of parameters used in each prediction model and characteristics of training cohorts

Model	Year	Number of patients	Procedure	Follow-up (in years)	Equation
Baltasar_1	2009	135	Duodenal switch	3	EBMI=0.33*IBMI+14
Baltasar_2	2011	7410	Any bariatric procedure	3	PBMI=IBMI*0.4+11.75
Baltasar_3	2011	2083	RYGB	3	PBMI=IBMI*0.4+10.23
Baltasar_4	2011	128	SG	3	PBMI=IBMI*0.4+10.88
Wood	2014	2608	RYGB	0.5-2	50th%tileBMI=36.71+0.7308*(IBMI—50)+0.02551*(age— 50)−0.906*(time—6)+0.04298*(time—6)^2^−0.00052*(time—6)^3^−0.00527*(IBMI—50)*(time—6)+0.001542*(age—50)*(time—6)
Wise	2016	647	RYGB	1	EBMIL=6.4*female gender-6.7*black race-1.2*BMI_o_-3.7*HTN-6*DM
Goulard	2016	197	SG	1	BMI=-3.597+0.621*BMI+0.135*age
Seyssel	2018	444	RYGB	1	WL=0.4*preoperative weight-0.21*age
Cottam_1	2018	371	SG	1	%EWL=140.9-0.731*DM-1.53*HTN-0.304*age-1.22*BMI-12.5*HTN*DM
Cottam_2	2018	371	SG	1	BMI reduction=0.73-0.0581*age+0.343*BMI-2.31*HTN*DM
Janik	2019	211	SG	1	InBMI=2.111+0.005*age+0.023*preoperative BMI+0.116*female gender
Velázquez-Fernández	2019	1002	RYGB	1	WL=-23.058+0.396*initial weight+0.035*days to visit+3.175*no sleep apnea

Abbreviations: *EBMI* expected body mass index, *IBMI* initial body mass index, *PBMI* predicted body mass index, *RYGB* Roux-en-Y gastric bypass, *SG* sleeve gastrectomy, *BMI* body mass index, *EBMIL* excess body mass index loss, *BMI*_o_, initial body mass index, *HTN* hypertension, *DM* diabetes mellitus, *WL* weight loss

### The Performance of Validated Models

According to the linear regression, all models presented significant relationship between predicted and actual postoperative BMI in ALL, RYGB, and SG groups. The closest correlation was achieved by Baltasar_2 model with regression coefficient 1.01, 1.00, and 1.00, respectively (Tables 4, 5, and 6).

**Table 4:** Results of linear regression analysis between predicted and observed BMI 1 year after surgery for prediction models in ALL group

Model	*B*	95% CI	*p*-value	*R*	*R*^2^	adjusted *R*^2^	SE	RMSE
Baltasar_1	1.22	1.05-1.39	**<0.0001**	0.46	0.21	0.21	5.13	5.13
Baltasar_2	1.01	0.87-1.15	**<0.0001**	0.46	0.21	0.21	5.13	5.13
Baltasar_3	0.94	0.81-1.07	**<0.0001**	0.46	0.21	0.21	5.13	5.13
Baltasar_4	0.94	0.81-1.07	**<0.0001**	0.46	0.21	0.21	5.13	5.13
Wood	0.58	0.50-0.66	**<0.0001**	0.47	0.22	0.22	5.11	5.10
Wise	0.29	0.25-0.33	**<0.0001**	0.43	0.18	0.18	5.21	5.20
Goulard	0.62	0.54-0.70	**<0.0001**	0.47	0.22	0.22	5.09	5.08
Seyssel	0.69	0.60-0.78	**<0.0001**	0.49	0.24	0.24	5.03	5.03
Cottam 1	0.56	0.49-0.64	**<0.0001**	0.46	0.21	0.21	5.13	5.12
Cottam 2	0.51	0.44-0.58	**<0.0001**	0.46	0.21	0.21	5.11	5.11
Janik	0.51	0.45-0.58	**<0.0001**	0.49	0.24	0.24	5.03	5.02
Velázquez-Fernández	0.67	0.58-0.76	**<0.0001**	0.47	0.22	0.22	5.10	5.09

*p*-value refers to linear regression coefficient

Embolden *p*-values indicate statistically significant result

Abbreviations: *B* regression coefficient, *95% CI* 95% confidence interval, *R* Pearson’s correlation coefficient, *SE* standard error of the estimate, *RMSE* root mean square error

**Table 5:** Results of linear regression analysis between predicted and observed BMI 1 year after surgery for prediction models in RYGB group

Model	*B*	95% CI	*p*-value	*R*	*R*^2^	adjusted *R*^2^	SE	RMSE
Baltasar_1	1.21	0.95–1.47	**<0.0001**	0.49	0.24	0.24	4.72	4.71
Baltasar_2	1.00	0.78–1.22	**<0.0001**	0.49	0.24	0.24	4.72	4.71
Baltasar_3	0.93	0.73–1.13	**<0.0001**	0.49	0.24	0.24	4.72	4.71
Baltasar_4	0.93	0.73–1.13	**<0.0001**	0.49	0.24	0.24	4.72	4.71
Wood	0.57	0.45–0.69	**<0.0001**	0.49	0.24	0.24	4.72	4.71
Wise	0.29	0.22–0.35	**<0.0001**	0.45	0.20	0.20	4.84	4.82
Goulard	0.59	0.45–0.72	**<0.0001**	0.47	0.22	0.22	4.78	4.76
Seyssel	0.66	0.52–0.80	**<0.0001**	0.50	0.25	0.25	4.70	4.69
Cottam 1	0.56	0.42–0.69	**<0.0001**	0.46	0.21	0.21	4.82	4.80
Cottam 2	0.50	0.38–0.61	**<0.0001**	0.47	0.22	0.22	4.79	4.77
Janik	0.50	0.39–0.61	**<0.0001**	0.49	0.24	0.24	4.72	4.71
Velázquez-Fernández	0.66	0.52–0.81	**<0.0001**	0.50	0.25	0.25	4.70	4.68

*p*-value refers to linear regression coefficient

Embolden *p*-values indicate statistically significant result

Abbreviations: *B* regression coefficient, *95% CI* 95% confidence interval, *R* Pearson’s correlation coefficient, *SE* standard error of the estimate, *RMSE* root mean square error

**Table 6:** Results of linear regression analysis between predicted and observed BMI 1 year after surgery for prediction models in SG group

Model	*B*	95% CI	*p*-value	*R*	*R*^2^	adjusted *R*^2^	SE	RMSE
Baltasar_1	1.22	1.00–1.44	**<0.0001**	0.44	0.19	0.19	5.31	5.30
Baltasar_2	1.00	0.82–1.19	**<0.0001**	0.44	0.19	0.19	5.31	5.30
Baltasar_3	0.21	0.17–0.24	**<0.0001**	0.44	0.19	0.19	5.49	5.49
Baltasar_4	0.93	0.77–1.10	**<0.0001**	0.44	0.19	0.19	5.31	5.30
Wood	0.58	0.48–0.69	**<0.0001**	0.45	0.20	0.20	5.28	5.27
Wise	0.29	0.23–0.34	**<0.0001**	0.41	0.17	0.17	5.39	5.38
Goulard	0.63	0.52–0.73	**<0.0001**	0.46	0.21	0.21	5.24	5.23
Seyssel	0.70	0.58–0.81	**<0.0001**	0.48	0.23	0.23	5.19	5.18
Cottam 1	0.56	0.46–0.66	**<0.0001**	0.45	0.20	0.20	5.29	5.27
Cottam 2	0.51	0.42–0.60	**<0.0001**	0.45	0.20	0.20	5.27	5.26
Janik	0.51	0.43–0.59	**<0.0001**	0.48	0.23	0.23	5.18	5.17
Velázquez-Fernández	0.67	0.55–0.79	**<0.0001**	0.45	0.20	0.20	5.27	5.26

*p*-value refers to linear regression coefficient

Embolden *p*-values indicate statistically significant result

Abbreviations: *B* regression coefficient, *95% CI*, 95% confidence interval, *R* Pearson’s correlation coefficient, *SE* standard error of the estimate, *RMSE* root mean square error

The two best predictive models in ALL group were Seyssel and Janik. They explained 24% variation of postoperative BMI (adjusted *R*^2^=0.24) and presented the best goodness-of-fit with lowest SE equaled 5.03 kg/m^2^. Detailed results of linear regression in ALL group are shown in Table 4.

In RYGB group, Seyssel and Velázquez-Fernández had the best predictive performance. Both models accurately foresaw 25% of BMI 1 year after the surgery (adjusted *R*^2^=0.25). The average difference between predicted and observed values was the lowest in abovementioned models and amounted to 4.70 kg/m^2^. Detailed results of linear regression in RYGB group are shown in Table 5.

Seyssel and Janik obtained the same best accuracy in SG group, explaining 23% variation of predicted BMI after intervention (adjusted *R*^2^=0.23). According to SE analysis, Seyssel presented slightly better calibration in comparison to Janik (5.19 kg/m^2^ vs 5.18 kg/m^2^). Detailed results of linear regression in SG group are shown in Table 6.

The worst predictive accuracy in all examined groups presented Wise model. It was able to predict from 17 to 20% of postoperative BMI values and in most cases reached supreme difference between predicted and actual BMI ranging from 4.84 to 5.39 kg/m^2^ (Tables 4, 5, and 6).

Comparison of mean predicted and observed BMI revealed that most of the models significantly overestimated achieved weight loss in all studied samples with the exception of Cottam_2, the only model underestimating outcome in all groups. The lowest differences between predicted and observed BMI in analyzed groups obtained Cottam_1 and Janik in the scope of 0.21–0.38 and 0.48–0.68, respectively. However, only Cottam_1 reached statistically significant goodness-of-fit, only in RYGB group (*p*=0.21). Detailed comparison between predicted and observed BMI is presented in Table 7 and Supplementary figure 1.

**Table 7:** Comparison of predicted and observed postoperative BMI in studied samples

Model	ALL			RYGB			SG		
	Predicted BMI	BMI difference	*p*-value	Predicted BMI	BMI difference	*p*-value	Predicted BMI	BMI difference	*p-*value
Baltasar_1	29.18±2.15	−3.64±5.15	<0.0001	29.30±2.20	−4.29±4.73	<0.0001	29.12±2.13	−3.30±5.32	<0.0001
Baltasar_2	30.15±2.61	−2.67±5.13	<0.0001	30.30±2.66	−3.30±4.71	<0.0001	30.08±2.58	−2.35±5.30	<0.0001
Baltasar_3	30.01±2.80	−2.81±5.13	<0.0001	30.16±2.86	−3.43±4.71	<0.0001	29.93±2.77	−2.49±5.31	<0.0001
Baltasar_4	30.66±2.80	−2.16±5.13	<0.0001	30.81±2.86	−2.78±4.72	<0.0001	30.58±2.77	−1.84±5.30	<0.0001
Wood	29.66±4.59	−3.16±5.45	<0.0001	30.03±4.67	−3.56±5.12	<0.0001	29.47±4.55	−2.95±5.60	<0.0001
Wise	25.35±8.49	−7.47±7.95	<0.0001	26.55±8.57	−7.04±7.78	<0.0001	24.73±8.39	−7.69±8.03	<0.0001
Goulard	30.75±4.38	−2.07±5.35	<0.0001	31.43±4.35	−2.16±5.09	<0.0001	30.40±4.36	−2.03±5.48	<0.0001
Seyssel	30.75±4.08	−2.08±5.18	<0.0001	31.18±4.09	−2.41±4.89	<0.0001	30.51±4.05	−1.91±5.32	<0.0001
Cottam_1	32.50±4.68	−0.32±5.51	0.007	33.38±4.48	−0.21±5.20	**0.21**	32.04±4.72	−0.38±5.67	0.02
Cottam_2	37.29±5.20	4.67±5.70	<0.0001	38.15±5.12	4.56±5.42	<0.0001	36.84±5.20	4.21±5.84	<0.0001
Janik	32.27±5.49	−0.55±5.69	0.0003	32.91±5.31	−0.68±5.40	0.04	31.94±5.55	−0.48±5.83	0.0055
Velázquez-Fernández	30.39±4.02	−2.43±5.26	<0.0001	30.62±4.07	−2.97±4.88	<0.0001	30.27±3.99	−2.15±5.42	<0.0001

Data are shown as mean ± standard deviation

BMI difference means the difference between predicted and observed body mass index after surgery

*p*-values refer to the comparison between predicted and observed body mass index after surgery with the use of a paired sample *t* test

Embolden p-values indicate statistically significant result

Abbreviations: *RYGB* Roux-en-Y gastric bypass, *SG* sleeve gastrectomy, *BMI* body mass index

## Discussion

Our study identified 12 models for the prediction of weight loss after bariatric surgery. Validation on independent cohort of 760 patients revealed significant correlation between predicted and observed BMI in all models. According to the accuracy, examined tools were able to explain from 17 up to 25% of the variation of weight loss outcome at 1 year. The majority of models significantly overestimated effect of bariatric treatment with the exception of Cottam_2. On average the predicted BMI was 4.70 to 5.49 kg/m^2^ lower than the actual.

Obtained findings confirmed bariatric surgery to be effective method of obesity treatment, reemphasized in numerous papers [1, 28, 29]. Implemented procedures resulted in significant postoperative weight loss followed by BMI reduction. More importantly, majority of patients achieved adequate effect of treatment with median EWL reaching 62.56%. This stays consistent with previously published studies reporting from 56 to 68% EWL depending on surgical procedure [30, 31].

The current scientific reports point that there is no homogenous weight loss curve after bariatric procedure for all patients [32]. Nevertheless, in all identified weight loss curves, distinct trajectories of initial weight change are followed by varied patterns of weight fluctuation over the longer term follow-up [32]. These findings suggest that the longer term outcomes in weight may be determined by the magnitude and direction or slope of the initial weight reduction. Therefore, preoperative estimation of 1-year outcome based on prediction models might enable to optimize early weight loss trajectory directions, consequently, providing better long-term outcome of bariatric treatment.

While there is an increasing awareness of the importance of prediction models and they are being published in increasing amount, there were hardly any attempts to provide their external validation and comparison of predictive performance. To our knowledge, only Sharples et al. performed such evaluation in 2017 including four models for weight loss prediction [14]. Since our study explored as many as 12 equations, it could provide more comprehensive, reliable, and up-to-date assessment of currently available weight loss prediction models.

In our research Baltasar_1 was able to explain 21% of postoperative weight, while previous validating studies presented better accuracy with the ability to predict outcome in 59% [33]. Although the prediction properties of Baltasar_3 and Baltasar_4 models were not reported in the original research, they were externally validated in independent paper, which revealed *R*^2^ equal 0.15 for Baltasar_3 and 0.34 for Baltasar_4 [34]. Present study finds comparable performance of these two models with similar *R*^2^ ranging from 0.19 to 0.21 depending on surgical procedure. Interestingly, by implementing both models into one cohort comprising patients after either RYGB or SG, Sharples et al. managed to obtain much higher accuracy with *R*^2^ value of 0.61 [33]. The same predictive performance Sharples et al. reported for Wood model [33]. Nevertheless, it differs greatly in comparison to our results (*R*^2^ 0.61 vs 0.22). Wise et al. was the first author who provided measures of fit along with model development [23]. According to the original study Wise was able to explain 35% of the variability of weight loss with average error of 17.4% EBMIL [23]. Present analysis demonstrated the worst accuracy of Wise model in all examined groups with the ability to predict only 17 to 20% of the outcome and one of the highest differences in estimation (RMSE from 4.84 to 5.39kg/m^2^). Goulard was the first model developed and validated on SG cohort [24]. Authors established good predictive accuracy of the model with *R*^2^ ranging from 0.61 to 0.76 as well as decent calibration with SE between 3.01 and 3.38 [24]. Nonetheless, our study revealed distinct outcomes with regard to the precision of Goulard’s estimations (*R*^2^=0.22 and SE=5.09kg/m^2^). Seyssel model in our analysis presented one of the highest *R*^2^ in all investigated cohorts ranging from 0.23 to 0.25 along with the lowest RMSE within 4.70 and 5.19 BMI points. Our results stay inconsistent with predictive properties provided in primary study. Not only training but also two independent validation cohorts suggested better accuracy with *R*^2^ equal to 0.45, 0.66, and 0.69, respectively, and RMSE ranging from 9.6 to 11.6 kg [25]. Cottam_1 and Cottam_2 models were able to explain respectively 39% and 27% of postoperative weight results at the time of formation, which is still better performance than 21% for both models obtained in our analysis [3]. Interestingly, Cottam_2 in our study presented better calibration than in original research (SE 5.11 kg/m^2^ vs 14.9 kg/m^2^) [3]. As RMSE was calculated in different units, we cannot make direct comparison of Wise, Seyssel, and Cottam_1 calibration between our analysis and original studies. During the creation, Janik model had accuracy comparable to Seyssel with ability to explain 67% of outcomes with only 0.12 kg/m^2^ difference from the actual values [26]. Nevertheless, in our population, it was able to predict 24% of the postoperative weight with higher error of 5.03kg/m^2^.

It is striking that all models explored in our analysis demonstrated considerably lower goodness-of-fit in comparison to the primary studies or previous validation researches. Possible explanation of such performance may be unstandardized surgical techniques. There are many technical differences in bariatric procedures including volume of the pouch or limb lengths in RYGB and distance from the pylorus, bougie size, or completeness of fundus resection in SG [35, 36]. As all of these procedural modifications and adjustments have significant impact on clinical outcomes, they may have affected comparison among different bariatric centers [35–37].

According to the comparison of predicted and observed postoperative BMI and differences between them, vast majority of examined models significantly overestimated ultimately achieved weight loss. Observed tendency may stem from baseline characteristic of the study group. The rates of comorbidities among patients seen in our cohort, particularly T2DM and HTN, are higher than those reported in the literature [38]. Chronic medical conditions are known to have significant impact on weight loss outcome after bariatric surgery [8]. Thus, they may have contributed to the overestimation of weight loss in our population.

Additionally, analysis of results in patients solely after RYGB or SG revealed better accuracy and calibration of all models in RYGB group than in SG group, even for tools originally dedicated to SG. Data shows that there is much wider variability in weight loss outcomes after SG in comparison to RYGB, which makes them more difficult to predict [1]. Consequently, this may have led to higher discrepancy in estimations and could explain observed differences.

The model proposed by Seyssel et al. achieved in our study the highest *R*^2^ value along with the lowest SE and modest difference between predicted and observed postoperative BMI, suggesting it to have the closest fit and be most likely to predict weight loss after bariatric surgery. Nevertheless, predictive accuracy of the model still remains at the insufficient level. As there is a wide range of previously mentioned factors influencing effects of bariatric treatment, not included into analyzed equations, it seems essential for further studies to incorporate these additional variables into prediction models so as to improve their accuracy [6–11].

It is worth noticing that there are other components affecting weight loss not easily measurable or impossible to obtain before surgery including eating habits, educational status, impulsivity, genetics, or gut hormone response [7, 9, 39, 40]. They are unavailable for the development of preoperative mathematical equation, resulting in limited predictive power of any preoperative model. *R*^2^ values presented in our research may be the bound of explanatory value of preoperative predictors as there are many other factors which play important role in the process of body weight reduction.

### Limitations

The study has several limitations. Firstly, due to its retrospective design, there is possible inconsistency of collected data. Secondly, as the study was undertaken in single center, the number of participants was relatively low. Although a number of patients in all samples were rather small for data extrapolations, they were adequately powered to provide reliable external validation of the models. Furthermore, our study comprised only Caucasian patients. As there is extensive evidence for weight loss variability among ethnical groups, it is unclear whether similar findings can be transmitted into worldwide population [7]. Further prospective validation with a larger sample size including more diverse population is needed to fully understand the efficacy of the prediction models and confirm ethnic differences.

Additionally, we only assessed results of 1-year follow-up which is insufficient to provide estimation of sustained weight loss. However, such timescale seems to be more useful, as the majority of weight loss after both SG and RYGB is achieved within the first year [1]. More importantly application of abovementioned criteria for the length of follow-up was necessary to provide accurate validation comparable with previous outcomes as majority of examined models were developed with the use of 1-year supervision. Moreover, setting 1-year observation enabled to avoid large drop-off in follow-up reported by other authors [22, 33]. Finally, other factors which may have an effect on weight loss such as development of postoperative complications and modification in physical activity or dietary behavior after surgery as well as alcohol intake, substance abuse, compliance to visits, psychological profiles, genetic background and support groups participation.

## Conclusion

In summary, our study identified 12 models for weight loss prediction after RYGB and SG, all of which have correlation with postoperative outcome. Seyssel model seem to have the best goodness-of-fit and utility as a prediction rule before surgery. However, the estimation should always be followed by physicians’ comment emphasizing that predicted outcome is only orientative and the final result depends on multiple factors. Further studies should focus on prospective assessment of available predictive models on larger, more diverse population and, if possible, improve their accuracy by including additional variables.

## Supplementary Information

ESM 1 (DOCX 13 kb)

ESM 2 (PNG 3607 kb)

High resolution image (TIF 422 kb)
