# Supplementary material for: Is It Possible to Predict Weight Loss After Bariatric Surgery?—External Validation of Predictive Models
Source: Obes Surg. 2021 Mar 13;31(7):2994–3004. doi: 10.1007/s11695-021-05341-w (PMC8175311; doi:10.1007/s11695-021-05341-w)
Supplement: Supplementary file 1 — (DOCX 13 kb) [file 11695_2021_5341_MOESM1_ESM.docx]

Supplementary table 1 BMI and weight change after surgery in studied samples

| ALL | | | |
| --- | --- | --- | --- |
| Parameter | Pre-op | Post-op | p-value |
| Weight, kg | 130.00 (30.00) | 94.79 (19.00) | **<0.0001** |
| BMI, kg/m^2^ | 46.00±6.52 | 32.82±5.76 | **<0.0001** |
| RYGB | | | |
| Parameter | Pre-op | Post-op | p-value |
| Weight, kg | 131.00 (30.50) | 96.79 (17.00) | **<0.0001** |
| BMI, kg/m^2^ | 46.36±6.65 | 33.59±5.40 | **<0.0001** |
| SG | | | |
| Parameter | Pre-op | Post-op | p-value |
| Weight, kg | 130.00 (29.00) | 92.79 (17.00) | **<0.0001** |
| BMI, kg/m^2^ | 45.81±6.44 | 32.42±5.90 | **<0.0001** |

Data are shown as mean ± standard deviation, median (interquartile range)
p-values refer to the comparison between parameters assessed pre- and postoperatively with the use of a paired sample t test or Wilcoxon test
embolden p-values indicate statistically significant result
Abbreviations: Pre-op, preoperative; Post-op, postoperative; BMI, body mass index; RYGB, Roux-en-Y gastric bypass; SG, sleeve gastrectomy;
