# Supplementary figures and images for: Is It Possible to Predict Weight Loss After Bariatric Surgery?—External Validation of Predictive Models
Source: Obes Surg. 2021 Mar 13;31(7):2994–3004. doi: 10.1007/s11695-021-05341-w (PMC8175311; doi:10.1007/s11695-021-05341-w)

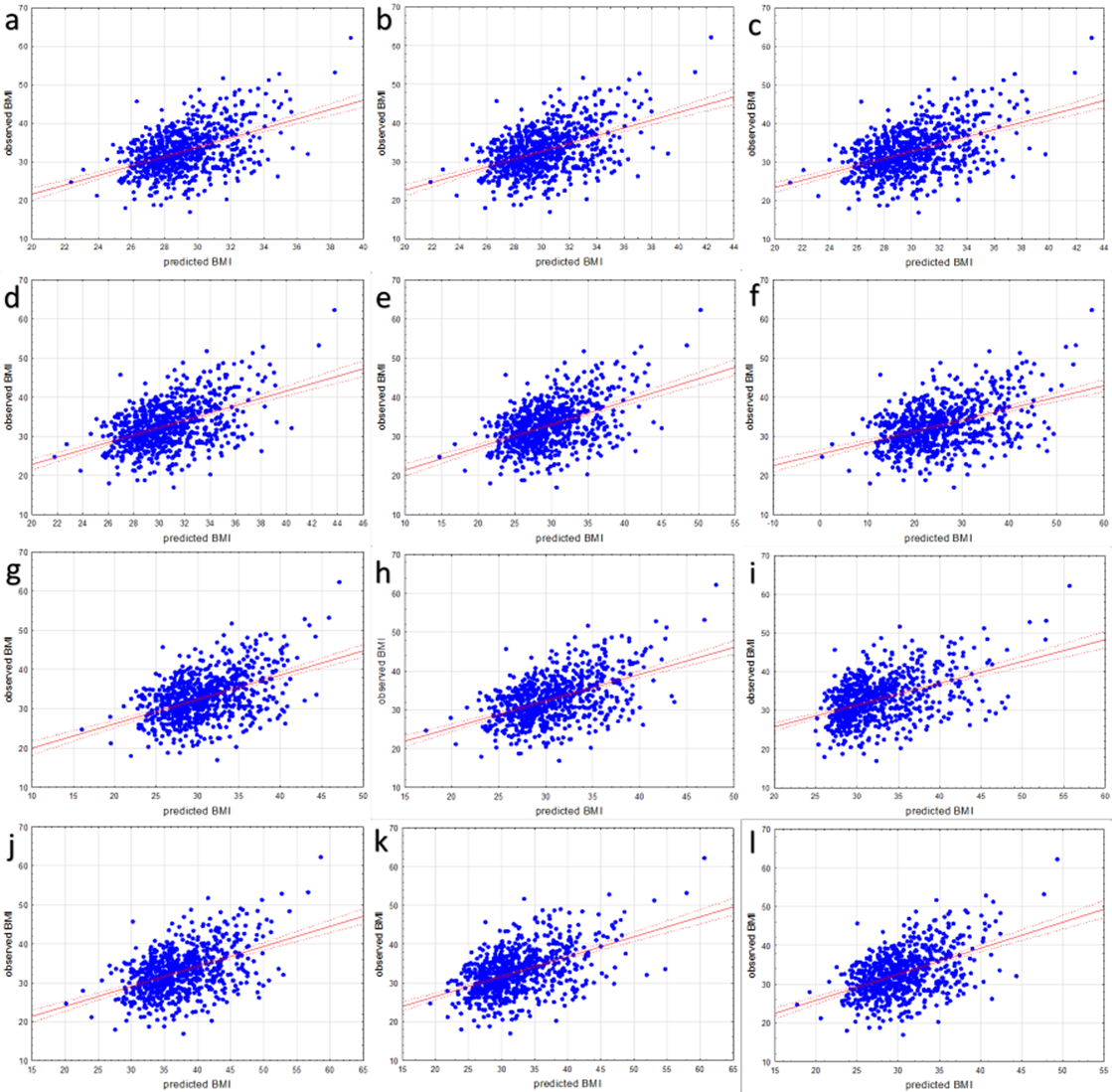

Supplement: Supplementary file 2 — (PNG 3607 kb) [file 11695_2021_5341_Fig2_ESM.png]

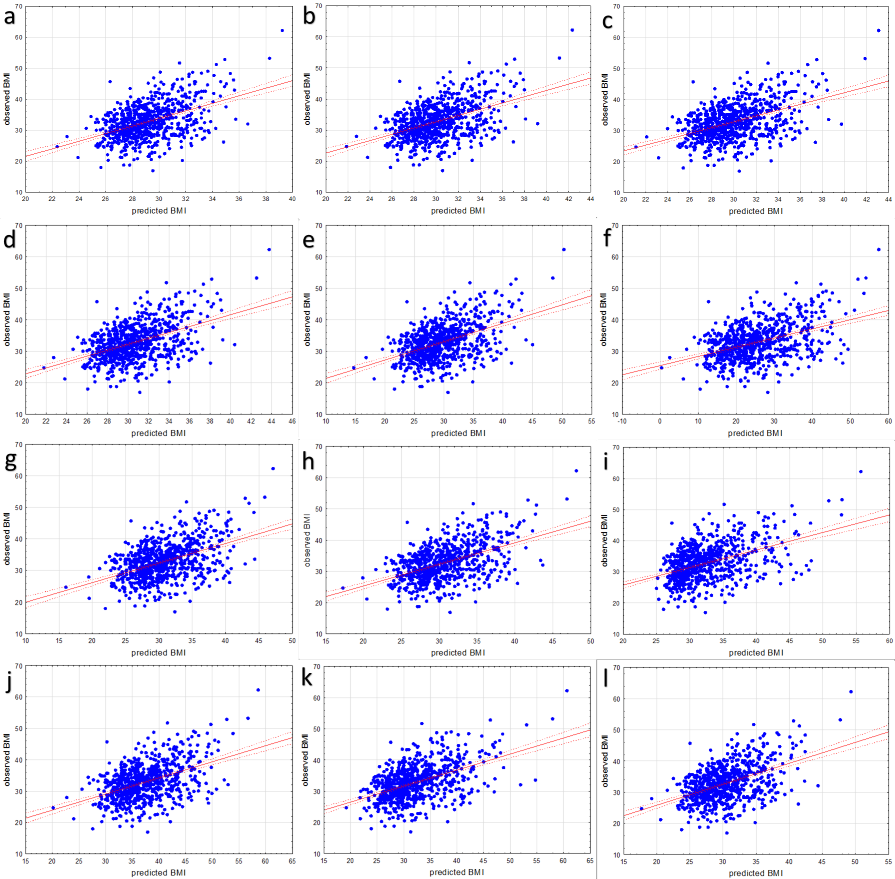

Supplement: Supplementary file 3 — High resolution image (TIF 422 kb) [file 11695_2021_5341_MOESM2_ESM.tif]
